# Influence of Naturally Occurring Bacteria on Embryonic and Larval Development of Common Toad Tadpoles

**DOI:** 10.3390/biology14030308

**Published:** 2025-03-19

**Authors:** Olga Jovanović Glavaš, Ines Sviličić Petrić, Goran Palijan

**Affiliations:** 1 Department of Biology, University of Osijek, Cara Hadrijana 8/A, 31000 Osijek, Croatia; ojovanovic@biologija.unios.hr; 2 Institute Ruđer Bošković, Bijenička Cesta 54, 10000 Zagreb, Croatia

**Keywords:** Amphibia, tadpoles, *Bufo bufo*, *Bacillus pumilus*, *Mesobacillus* sp., biomarkers

## Abstract

Amphibians, as the most threatened group of vertebrates, are the focus of investigation of various agents that could affect their fitness and survival. In this context, we examined the effects of naturally occurring bacteria and their combinations on the embryonic and larval development of common toad tadpoles (*Bufo bufo*). One egg string that was collected from the wild was disinfected in the lab and divided into short strings, each containing 20 eggs. These strings were exposed to three different control treatments, i.e., a sterile FETAX solution, water collected from the tadpoles’ native habitat, and sterilized habitat water, as well as to three different bacterial cultures isolated from habitat water (*Bacillus* sp., *Mesobacillus* sp.). We analyzed several morphometric variables (snout–vent length, total length, and weight), the energy budget by measuring body composition (proteins, carbohydrates, and lipids), and biomarker activity (acetylcholinesterase and lactate dehydrogenase). Our results indicate that the native microbial community had a negative effect on egg and tadpole development, as sterilized habitat water supported their highest development. Additionally, when grown in FETAX, pure bacterial cultures and their combinations decreased acetylcholinesterase activity but positively affected both tadpole size and energy budget. These findings suggest that bacteria can influence the larval development of common toads by modifying physiological traits. Future research should identify which microbes have beneficial or detrimental effects on amphibian development.

## Simple Summary

To investigate the impact of bacteria on the embryonic and larval development of the common toad (*Bufo bufo*), eggs and tadpoles were exposed under laboratory conditions to bacteria (*Bacillus* sp., *Mesobacillus* sp.) isolated from the habitat water. Pure bacterial cultures and their combinations decreased acetylcholinesterase activity but had positive effects on tadpole size and energy budget. These findings suggest that bacteria can influence the larval development of common toads by modifying physiological traits. Future research should elucidate which microbes have beneficial or detrimental effects on amphibian development.

## 1. Introduction

Bacteria are ubiquitously present in nature, persisting across both space and time. They pervade the biosphere, inhabiting environments ranging across oceans and ocean sediments, the lithosphere, and terrestrial and freshwater ecosystems. In all of these habitats, bacteria interact closely with other organisms, some of which form very strong connections with bacteria. For instance, plant-growth-promoting bacteria enhance plant growth and health under various environmental conditions [1]. In other cases, bacteria form obligate endosymbiotic relations with many arthropods and nematodes [2]. Nevertheless, research on bacterial interactions with vertebrates, other than humans or livestock, is still gathering pace [3], although the importance of this relationship has been especially recognized in recent decades [4]. Still, microorganism–amphibian interactions are largely underrepresented and understudied in the scientific literature [5].

Animal–bacterial interactions are gaining increasing attention due to the recognition of their profound impact, ranging from the influence of the human microbiome on human health [6] to the stimulation of settlement and metamorphosis in marine invertebrate larvae [7]. Tadpoles serve as a good model system for studying vertebrate responses to environmental factors. They are often used to assess the effects of various agents on amphibians, including pesticides [8], metals in bulk and nano form [9], and microplastic pollution [10]. In addition to the effects of chemical pollutants, different aspects of tadpole–bacteria interactions were investigated. Jones et al. [11] recognized the importance of colonization order of native bacteria isolates on the microbiome structure of hourglass treefrog tadpoles, *Dendropsophus ebraccatus*. Furthermore, the developmental stage has been shown to have a much stronger effect on microbiome structure than temperature variations [12]. Similarly, Santos et al. [13] demonstrated that the skin and gut microbiomes of *Pelophylax perezi* and *Bufo spinosus* vary depending on both the host species and the water environment. Moreover, the gut microbiome was negatively impacted by glyphosate-based herbicides and antibiotics in *Rana berlandieri* tadpoles [14]. The impact of pollutants on tadpole microbial communities could result in indirect negative effects on tadpoles, as the interaction between tadpoles and their microbiome is known to be beneficial for their development [15].

Bacteria, as the most widespread microorganisms, exhibit tremendous species richness, providing vast opportunities for interactions. Young animals acquire microbes from their immediate environment. In the case of tadpoles, they interact with microbes from the aquatic environment but also with those that have washed in from the surrounding soil. One common bacterial group in these habitats is the genus *Bacillus*. These Gram-positive, sporulating microbes contain diverse metabolic, physiological, and ecological traits, such as the production of lipopeptide, antibiotic, hydrogen cyanide, siderophore, extracellular hydrolytic enzymes, and toxins, as well as nitrogen fixation, phosphate solubilization, and phytohormone synthesis [16]. These properties enable them to induce systemic resistance in plants, enhance their health and growth inhabiting the rhizosphere or as endophytes [1,17], and suppress pathogenic fungi [18]. They also colonize the gut of black tiger shrimp, where they help protect from pathogens [19], and some members of the genus are even considered probiotics [20]. Given these characteristics and their effects on plants and invertebrates, it is reasonable to ask whether similar interactions could influence other animals, such as amphibians.

Amphibians are terrestrial vertebrates characterized by highly permeable skin that interacts with microorganisms from the environment, creating a unique microbiome. Its role is very versatile, from maintaining surface integrity to protecting the organisms from numerous pathogens such as the fungus *Batrachochytrium dendrobatidis* or ranaviruses [21,22,23]. Amphibians are the most threatened group of vertebrates, with over 40% of all species being threatened with extinction; moreover, diseases account for one-third of the total threats facing them [24]. However, despite the critical importance of microbial–skin interactions for amphibian health, most studies refer to various pathogens, and very few studies are available on other roles of microbial–skin interactions.

The common toad, *Bufo bufo* (Linnaeus, 1758) is a widespread European species, but in most habitats it is not very abundant. It tends to breed in it natal breeding site, and is therefore more susceptible to any changes occurring within the site [25].

Acetylcholinesterase (AChE, EC 3.1.1.7) is an enzyme that catalyzes the hydrolysis of the ester bond of acetylcholine, the most important neurotransmitter. It is one of the fastest enzymes with a unique molecular structure [26], and various stressors can induce or inhibit its activity. Despite its physiological significance, AChE activity has only occasionally been measured in tadpoles. For example, Johnson et al. [27] examined the impact of different water temperatures on AChE activity, while Attademo et al. [28] reported statistically significant inhibition of AChE by microplastics and plastic additives. However, no effect was observed after exposure to pesticide metaldehyde [29].

Lactate dehydrogenase (LDH, EC 1.1.1.27) is a notable enzyme of the anaerobic metabolic pathway. It catalyzes a reversible reaction in which lactate is converted to pyruvate accompanied by the reduction of NAD^+^ to NADH, and vice versa [30]. Variations in lactate dehydrogenase activity usually indicate some metabolic changes. In amphibians, changes in LDH activities normally occur during the ontogenetic development of embryos, and stabilize later in the tadpole stage [31]. In adult amphibians, changes in LDH activity are observed in response to intensive physical activities, and exposure to stress, e.g., pH and temperature [32,33].

In the present study, we examined the effects of three pure bacterial cultures isolated from the tadpoles’ habitat, as well as combinations of them, on the growth and physiological status of common toad tadpoles. To the best of our knowledge, this is the first time that the impact of bacteria on toad embryonic and larval development has been reported. We hypothesized that bacterial exposure would significantly affect tadpole development. To test this hypothesis, the energetic budget and physiological status of tadpoles were measured.

## 2. Materials and Methods

### 2.1. Isolation and Characterization of Bacteria

Tadpoles and water samples were collected from Lake Borovik in eastern Croatia (GPS coordinates 45°23′29.6″ N 18°10′36.2″ E). Water sample was collected on 9 March 2019 using sterilized jar (1 L) and analyzed within 24 h after collection. It was spread plated after serial dilution on nutrient agar (Biolife, Milano, Italy) and incubated at 22 ± 1 °C. Three randomly chosen separated colonies were isolated and preserved for future use on agar slants and in glycerol at −80 °C (Binder, Tuttlingen, Germany).

DNA was extracted from pure colonies of three isolates grown on the nutrient agar plates using the Quick-DNA Miniprep Plus Kit (Zymo Research, Freiburg, Germany). The 16S rRNA marker gene was amplified via PCR (machine PCRmax Alpha Cycler 1, Cole-Parmer, Vernon Hills, IL, USA) using universal primers 27F and 1492R [34] with the following program: 95 °C for 5 min, 30 cycles of 95 °C for 45 s, 55 °C for 1 min, 72 °C for 1.5 min, and a final extension at 72 °C for 10 min. Obtained PCR products were purified and Sanger-sequenced by Macrogen (Amsterdam, The Netherlands). The obtained raw sequences were edited with Chromas Lite v2.6.6 (Technelysium, South Brisbane, Australia) and analyzed using BLASTn 2.16.1+ against the NCBI database.

### 2.2. Experimental Design

The microcosmic experiment was conducted in heat-sterilized 720 mL round glass jars. Each jar was filled with 500 mL sterile FETAX solution (625 mg NaCl, 96 mg NaHCO_3_, 75 mg MgSO_4_, 75 mg CaSO_4_·2H_2_0, 30 mg KCl, 15 mg CaCl_2_ per liter distilled water). One string of *Bufo bufo* eggs was collected from the field on 18 March 2019 and kept in the fridge (4 °C) in a jar filled with lake water until experimental setup the following day. Prior to use, the string was disinfected by submerging the eggs into 70% ethanol for 20 s and rinsing three times in sterile distilled water. In each jar, a string of 20 sterilized eggs (Gosner stage (GS) 11 [35]) was added. After that, each treatment received 1 mL of overnight bacterial culture adjusted to OD_600_ = 1, or an equal mixture of two cultures, or all three cultures of a total volume of 1 mL. The solution of sterile FETAX without added bacteria was the control (K1). Besides K1, there were two additional controls: one consisting of non-sterile lake water (K2), and the second consisting of autoclaved lake water (K3). These additional controls were taken to assess the suitability of FETAX for tadpole development and to investigate the effects of native microbial communities present in K2 but absent in K3. All jars were on a shelf without direct exposure to sunlight and closed with screw caps. The microcosms were not aerated, but each cap had a mounted 0.2 μm pore size syringe filter.

All treatments were carried out in triplicate. Microcosms were kept at room temperature (22 ± 1 °C) and in a natural light cycle for two weeks. The order of the jars was random, and their position was changed daily. Upon hatching, which occurred after two to three days, all tadpoles were fed daily ad libitum with sterilized commercial fish food (Tubifex, Vitakraft, Bremen, Germany). The medium was not changed during the experiments. After two weeks, from each flask, three tadpoles were randomly chosen (i.e., nine tadpoles per treatment), weighted (precision 0.001 g), had their snout–vent length (from the tip of the snout to the most posterior opening of the cloacal slit; SVL) and tail length (from the posterior edge of the body to the tip of the tail fin; TL) measured using a hand caliper (precision 0.01 mm), and, together with other tadpoles, were frozen at −80 °C until further analysis (some of which are not included in this paper). The experiments were conducted in accordance with the EU legislation for animal experimentation. Eggs that were not used in the experiment were returned to the site where they had been collected.

### 2.3. Molecular Biomarkers and Energy Budget Analyses

Whole tadpoles were homogenized in phosphate buffer (0.1 M, pH 7.4; ratio 1:5 *w*/*v*). Protein content was determined according to the methodology detailed by Bradford [36], homogenate: buffer 1:3, at 595 nm. Total lipid content was determined by applying the phospho-vanillin protocol [37]. Total carbohydrate content was calculated using the spectrophotometric anthrone protocol [38]. Subsequently, the homogenates were centrifuged at 9000× *g* for 30 min at 4 °C, and supernatants were used for further analysis. The activity of LDH was determined using the protocol set out in [39], and then measured for 60 s at 340 nm. The activity of AChE was measured for 30 s at 412 nm and calculated according to protocol detailed by Ellman et al. [40].

### 2.4. Statistical Analysis

Before analysis, data were checked for normality of distribution. The Kolmogorov–Smirnov test showed that TL, weight, and AChE did not follow normal distribution and were log x + 1-transformed. Variables were analyzed between treatments via Bayesian one-way ANOVA, after which the post hoc test was used (prior vs. posterior odds) to detect which bacterial treatments differed from the control (K1). The posterior odds were corrected for multiple testing; hence, it is a conservative measure. Bayesian ANOVA does not use one of the factors as reference, but instead calculates the total mean and then compares all other means to the total mean. In this way, the analysis does not depend on the *p*-value, which is an arbitrary chosen value, and thus subjective, but instead depends on the intrinsic nature of the data set itself. Also, the Bayes factor provides hypothesis testing in terms of probability, which offer continuous conclusions as probability could be anywhere between low and high, while the *p*-value-based significance test is only dichotomous in the sense that we can only conclude whether the difference exists (on the arbitrary threshold) or not, but without any gradation. The Bayes factor (BF_10_) was calculated for the null hypothesis of no difference between the treatments and for the alternative hypothesis, both of which were considered equally likely. Also, the robustness of analysis was calculated as a error percentage. This value should be below 20%, with lower values indicating a greater numerical stability of the results [41]. Bayesian tests were conducted in Open Source software JASP 0.19.3.

## 3. Results

Bacterial isolates were identified as *Mesobacillus* sp. (culture A) or *Bacillus pumillus* (culture B), while the third culture (culture C) was left unidentified due to difficulties in sample preparation.

For all tested variables except TL and proteins, the alternative hypothesis was more likely (Table 1), suggesting the existence of differences between the treatments.

Post hoc tests resulted in prior odds of 0.149 for all comparisons. Hence, all posterior odds larger than 0.149 represent a higher probability of effect.

**Figure 1:**
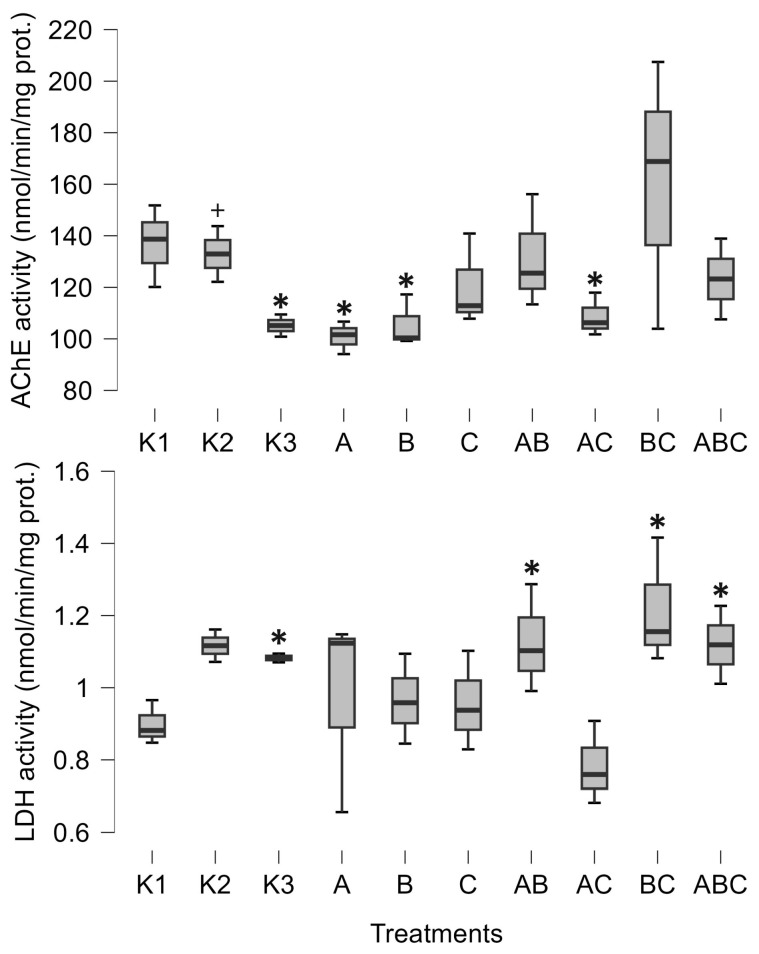
Minimum, maximum (whiskers), and median (bar) values of tadpoles’ acetylcholinesterase activity (AChE) and lactate dehydrogenase activity (LDH). The asterisk marks significant differences compared to K1, while the plus marks a significant difference compared to K3. K1—sterile FETAX solution, K2—lake water, K3—sterile lake water, A—*Mesobacillus* sp., B—*Bacillus pumilus*, C—unidentified bacteria.

The pure culture of *Mesobacillus* sp. significantly decreased AChE activity (posterior odd 0.465, Figure 1). AChE activity was also significantly decreased when exposed to the pure culture of *Bacillus pumilus* (posterior odd 0.301, Figure 1) and with a combination of *Mesobacillus* sp. and unidentified bacteria (posterior odd 0.266, Figure 1). Lactate dehydrogenase was increased as a result of the combinations of *Mesobacillus* sp. and *B. pumilus*, *B. pumilus* and unidentified bacteria, and through the combination of all three cultures (posterior odds 0.246, 0.332 and 0.216, respectively, Figure 1).

**Figure 2:**
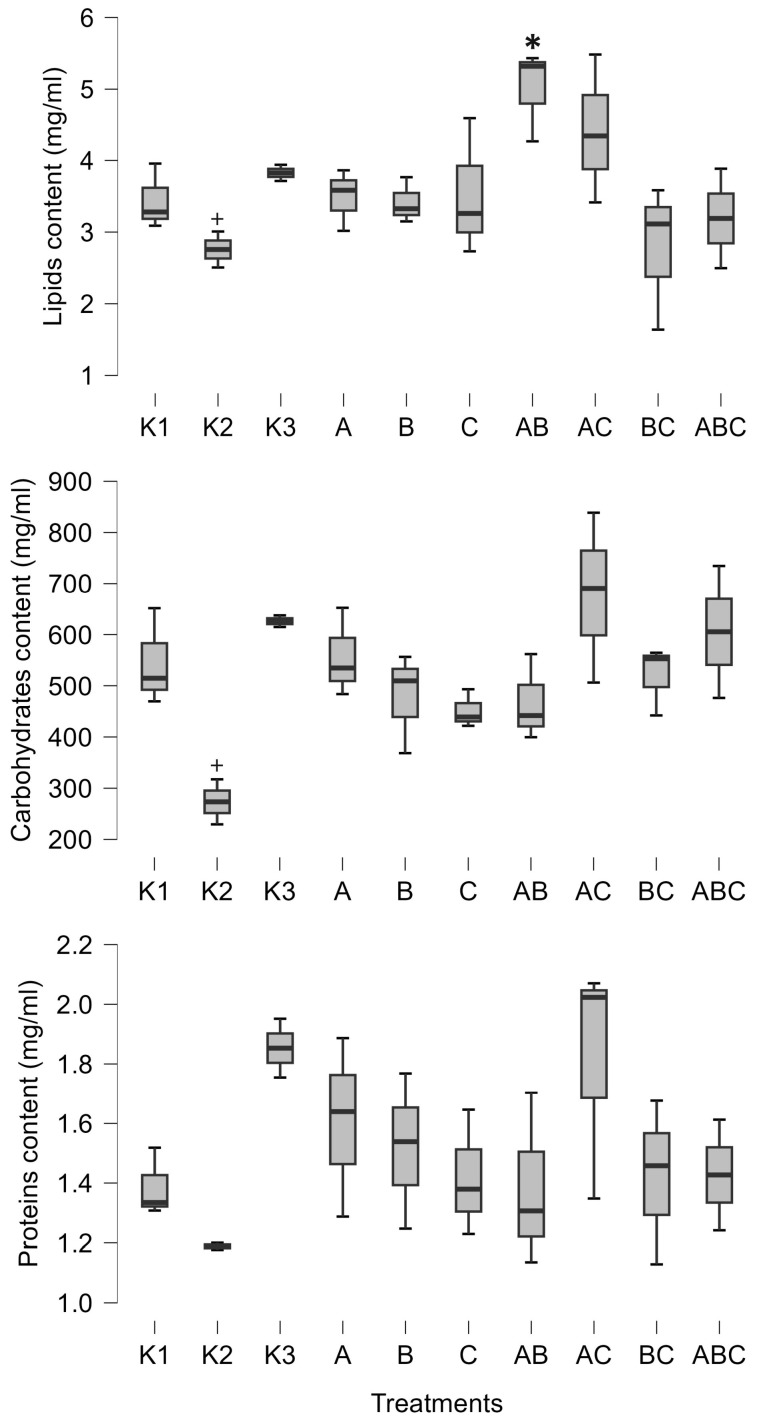
Minimum, maximum (whiskers), and median (bar) values of tadpoles’ lipids, carbohydrates (C-H), and protein content. The asterisk marks significant differences compared to K1, while the plus marks a significant difference compared to K3. K1—sterile FETAX solution, K2—lake water, K3—sterile lake water, A—*Mesobacillus* sp., B—*Bacillus pumilus*, C—unidentified bacteria.

**Figure 3:**
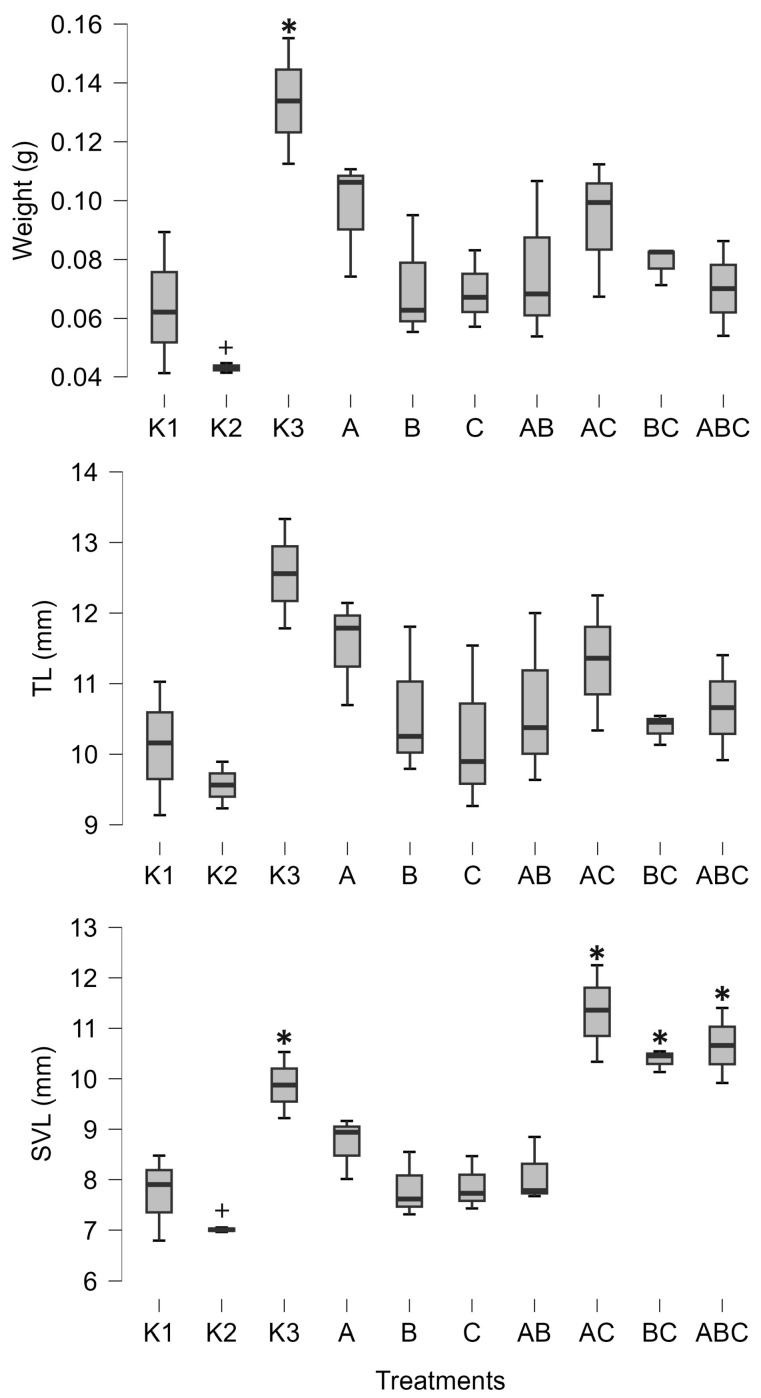
Minimum, maximum (whiskers), and median (bar) values of tadpoles’ weight, tail length (TL), and snout–vent length (SVL). The asterisk marks significant differences compared to K1, while the plus marks a significant difference compared to K3. K1—sterile FETAX solution, K2—lake water, K3—sterile lake water, A—*Mesobacillus* sp., B—*Bacillus pumilus*, C—unidentified bacteria.

Furthermore, the combination of *Mesobacillus* sp. and *Bacillus pumilus* significantly increased the amount of lipids in tadpoles (posterior odd 0.425, Figure 2). SVL was positively influenced by the combinations of *Mesobacillus* sp. and *B. pumilus* with unidentified bacteria, as well as by the combination of all three cultures (posterior odds 0.846, 0.996, and 0.34, respectively, Figure 3). Although weight and carbohydrates appear to increase as a result of the combination of *Mesobacillus* sp. and the unidentified bacteria, this was not detected by post hoc analysis as posterior odds were smaller compared to prior.

The control treatments for the effect of natural microbial communities suggest a negative impact of the aquatic microbial community on tadpole development. Specifically, K2 (non-sterile lake water) significantly decreased weight (posterior odd 0.317, Figure 3), SVL (posterior odd 0.315, Figure 3), carbohydrates (posterior odd 0.523, Figure 2), and lipids (posterior odd 0.286, Figure 2), while AChE activity was increased (posterior odd 0.212, Figure 1) compared to K3 (sterile lake water). LDH activity remained unchanged.

Tadpoles were larger in sterile lake water (K3) compared to FETAX (K1). LDH, weight, and SVL had higher values in K3 (posterior odds 0.409, 0.27, and 0.246, respectively, Figure 1 and Figure 3). AChE activity was decreased in lake water (K3) (posterior odd 0.234, Figure 1). Lipid and carbohydrate levels did not differ between sterile lake water (K3) and FETAX (K1), although they had slightly higher values in K3 (Figure 2).

The microcosms were not probed for bacteria at the end of the experiment. This means that there is a possibility that other bacteria, which survived disinfection, developed during the two-week incubation period alongside the added treatments. Nevertheless, these bacteria were certainly present in substantially lower abundance compared to the introduced bacteria. Therefore, their impact, if any, was probably minimal.

**Figure 4:**
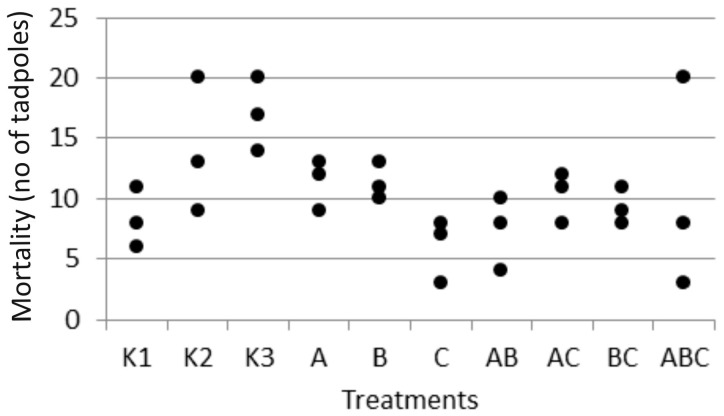
Mortality occurred during the experiment as number of dead tadpoles in each triplicate of the treatment. There was no difference between groups grown in FETAX (Bayesian ANOVA).

Finally, mortality rates observed during the experiment (Figure 4) did not differ significantly between the groups with FETAX (Bayesian ANOVA), suggesting that it was not related to experimental treatments, i.e., added bacteria. In lake water controls, mortality was highest, especially in the sterile lake water (K3) (Figure 4).

## 4. Discussion

In this paper, we describe the interactions between *Bufo bufo* tadpoles and *Bacillus pumilus* and *Mesobacillus* sp. One important finding of our study was a decrease in AChE activity induced by *Mesobacillus* sp. and *B. pumilus* pure cultures, as well as in combination with *Mesobacillus* sp. with unidentified bacteria. The activity of AChE is an indicator of environmental stress, such as temperature or exposure to xenobiotics [27,28]. In our study, a significant decrease in AChE activity was observed solely due to the presence of different bacteria. On the other hand, the native microbial community (K2) increased AChE activity compared to sterilized lake water (K3). This is an important finding as it suggests that different members of the aquatic microbial community can either increase or decrease AChE activity in tadpoles. So far, we have confirmed that *Mesobacillus* sp. and *B. pumilus* decrease its activity. This enzyme is responsible for the normal functioning of neuromuscular junctions, and its inhibition can lead to muscular paralysis. In our study, AChE activity was not completely inhibited, but was decreased. In such circumstances, it is possible that tadpoles were less active and hence able to accumulate biomass. Our results support this idea, as decreased AChE activity was accompanied by an increase in tadpoles’ size and energetic status (Figure 2 and Figure 3). *Mesobacillus* sp. is one of the new genera derived from *Bacillus*. Members of this genus are aerobic or facultatively anaerobic, Gram-positive, rod-shaped microbes inhabiting different environments including soil [42] and freshwater lake sediment [43]; however, further information is scarce [44]. On the other hand, *B. pumilus* is a well-studied bacterial species, particularly in the context of plant growth promotion [1]. In plant interactions, these bacteria provide protection against fungal phytopathogens [18], produce phytohormones [45], and synthesize ACC deaminase [46], supporting plant development through the rhizosphere [18] or as endophytes [47]. Murugappan et al. [47] found increased root and shoot length, as well as an increased number of leaves, in plants inoculated with *B. pumilus*. Furthermore, *B. pumilus* induces systemic resistance in plants, indicating its ability to modulate the plant immune system [48]. There is a wide surface through which *B. pumilus* interacts with plants, and it is highly probable that such a wide span of interactions also exists with animals [4]. Dökenel and Özer [49] detected *B. pumilus* on marsh frogs (*Pelophylax ridibundus*) from a frog farm, but not in water or feed samples. This result suggests that *B. pumilus* exhibits some degree of association with at least one frog species. Similarly, Scalvenzi et al. [5] reported the constant presence or dominance of *Firmicutes* (including the *Bacillus* genus) in different developmental stages of *Xenopus tropicalis*.

Our results suggest that *B. pumilus* and *Mesobacillus* sp. may influence tadpole physiology. Furthermore, the interaction between *B. pumilus* and unidentified bacteria appears to mitigate the negative effect of *B. pumilus* on AChE activity. Moreover, the overall microbial community significantly increased AChE activity, highlighting the importance of microbial interactions in their effects on tadpoles [50]. AChE activity is seldomly measured in tadpoles and serves as an indicator of chemical environmental contamination. Attademo et al. [29] assessed the effects of metaldehyde on AChE activity in common toad (*Rhinella arenarum*) tadpoles but found no significant effects, similar to the lack of effects observed with the use of glufosinate-ammonium-based herbicide on tadpoles of the same species [51]. Conversely, the same authors recorded an increase in AChE activity due to glyphosate-based herbicides [51]. However, Attademo et al. [28] noted a significant decrease in AChE activity caused by polyethylene microplastics (40–48 µm particle size, 60 mg L^−1^) and by plastic additive tetrabromobisphenol A in tadpoles of the same species. In their investigation, AChE activity decreased by approximately 25% compared to the control, which is comparable to our findings. Depending on the treatments (pure culture or mixture), our work resulted in a 25% to 35% decrease in AChE activity. This suggests that both chemical pollution and bacteria present in the environment are equally important in shaping the physiological response of tadpoles. Nevertheless, we would like to emphasize that we used only one string of eggs in our experiment; hence, the inclusion of more egg strings would more precisely describe the variability in bacteria–tadpole interactions. Finally, LDH activity was stimulated by a combination of *B. pumilus* and unidentified bacteria, which is the same mixture that mitigated the negative effect of *B. pumilus* on AChE activity. Therefore, unidentified bacteria, which had no impact on AChE or LDH activity in pure culture, in combination with *B. pumilus* mitigated its negative effect on AChE activity and induced an increase in LDH activity. This emergent situation greatly complicates the investigation of host–microbe interactions, as their effects are altered by the development of microbe–microbe interactions. Due to the tremendous species richness of bacteria, a myriad of underlying interactions could exist. The nature of these microbe–microbe interactions, which differentially impact amphibians, remains to be discovered.

## 5. Conclusions

Our results demonstrate that bacteria present in the tadpole environment play a significant role, under laboratory conditions, in embryonic and larval development by modulating their physiology. Exposure of common toad (*Bufo bufo*) tadpoles to the whole microbial community resulted in increased AChE activity and decreased weight, SVL, lipids, and carbohydrates compared to sterile lake water. Conversely, the exposure of eggs and tadpoles to *Mesobacillus* sp. and *B. pumilus*, or unidentified bacteria, resulted in decreased AChE activity, while LDH, SVL, and lipids increased. However, these findings should be taken with caution, as tadpoles in their natural environment are not exposed to pure bacterial cultures or simple combinations thereof, but rather to complex microbial communities. Future research should perhaps focus on investigating the effects of different community structures.

## Figures and Tables

**Table 1:** Bayesian ANOVA output with prior and posterior odds, Bayes factors, and stability of the modes.

Models	P (M)	P (M|Data)	BF_10_	Error %
Null model	0.5	0.434	1	
AChE	0.5	0.566	1.306	3.386 × 10^−5^
Null model	0.5	0.393	1	
LDH	0.5	0.607	1.542	2.694 × 10^−5^
Null model	0.5	0.344	1	
Weight	0.5	0.656	1.906	2.556 × 10^−5^
Null model	0.5	0.579	1	
TL	0.5	0.421	0.728	8.520 × 10^−5^
Null model	0.5	6.828 × 10^−4^	1	
SVL	0.5	0.999	1463.601	2 × 10^−3^
Null model	0.5	0.305	1	
Lipids	0.5	0.695	2.278	1.594 × 10^−5^
Null model	0.5	0.31	1	
Carbohydrates	0.5	0.69	2.223	1.968 × 10^−5^
Null model	0.5	0.624	1	
Proteins	0.5	0.376	0.603	1.079 × 10^−4^

## Data Availability

The original contributions presented in this study are included in the article. Further inquiries can be directed to the corresponding author.
